# Dietary intake and diabetic retinopathy: A systematic review

**DOI:** 10.1371/journal.pone.0186582

**Published:** 2018-01-11

**Authors:** Mark Y. Z. Wong, Ryan E. K. Man, Eva K. Fenwick, Preeti Gupta, Ling-Jun Li, Rob M. van Dam, Mary F. Chong, Ecosse L. Lamoureux

**Affiliations:** 1 Singapore Eye Research Institute, Singapore National Eye Center, Singapore; 2 Duke-NUS Medical School, Office of Clinical Sciences, Singapore; 3 Singapore Institute for Clinical Sciences, A*STAR, Singapore; 4 Yong Loo Lin School of Medicine, National University of Singapore, Singapore; 5 Saw Swee Hock School of Public Health, National University of Singapore, Singapore; University of Tasmania, AUSTRALIA

## Abstract

**Introduction:**

The evidence linking dietary intake with diabetic retinopathy (DR) is growing but unclear. We conducted a systematic review of the association between dietary intake and DR.

**Methods:**

We systematically searched PubMed, Embase, Medline, and the Cochrane Central register of controlled trials, for publications between January 1967 and January 2017 using standardized criteria for diet and DR. Interventional and observational studies investigating micro- and macro-nutrient intakes; food and beverage consumptions; and dietary patterns were included. Study quality was evaluated using a modified Newcastle-Ottawa scale for observational studies, and the Cochrane collaboration tool for interventional studies.

**Results:**

Of 4265 titles initially identified, 31 studies (3 interventional, 28 Observational) were retained. Higher intakes of dietary fibre, oily fish, and greater adherence to a Mediterranean diet were protective of DR. Conversely, high total caloric intake was associated with higher risk of DR. No significant associations of carbohydrate, vitamin D, and sodium intake with DR were found. Associations of antioxidants, fatty acids, proteins and alcohol with DR remain equivocal.

**Conclusions:**

Dietary fibre, oily fish, a Mediterranean diet and a reduced caloric intake are associated with lower risk of DR. Longitudinal data and interventional models are warranted to confirm our findings and better inform clinical guidelines.

## Introduction

Diabetic retinopathy (DR) is a major microvascular complication of diabetes and a leading cause of vision loss and blindness globally[1]. Nearly all patients with type 1 diabetes and >60% of patients with type 2 diabetes will have some form of DR within 20 years of developing diabetes[2, 3]. With the rapidly increasing prevalence of diabetes worldwide, the prevention and management of DR has become a crucial public health concern[4].

Optimal nutrition forms a crucial component of overall diabetes care[5, 6]. While comprehensive dietary guidelines for overall diabetes management have been developed, these guidelines do not extend specifically to the prevention and management of DR[7, 8]. As such, DR-specific dietary recommendations for patients with diabetes at risk of development or progression of DR are not available. Several studies have explored the association between various dietary components and DR; they include micronutrients (e.g. antioxidants, sodium, vitamin D)[9–11], macronutrients (e.g. carbohydrates, proteins, fats)[12–14], food groups and beverages (e.g. fruit and vegetables, fish, coffee, tea)[15–18], as well as broader dietary patterns and characteristics (e.g. Mediterranean [Med] diet, total caloric intake)[10, 19]. However, findings remain inconclusive, and current evidence do not inform the specific dietary components which are likely to reduce (or increase) the risk of DR.

There are also few comprehensive reviews on diet and DR, with existing reviews mostly either focused on a specific nutrient or food group (e.g. alcohol, micronutrients)[20–22] or providing only a summary of the potential of the diet to influence DR pathogenic mechanisms. To our knowledge, there are no comprehensive review of the entire spectrum of dietary components and their association or effects on DR as a clinical outcome[23]. To address this major clinical gap, we performed a systematic review on the associations between dietary intake and DR, with the primary goal of providing a comprehensive assessment of the existing knowledge on the topic. We also identified key knowledge gaps and suggest future research directions.

## Methodology

### Literature search

No existing protocol exists for this systematic review. We performed a systemic review and comprehensive literature search using four databases (PubMed, Embase, Medline and the Cochrane Central Register of Controlled Trials), with a date range of January 1967 to January 2017 with no language restrictions. The databases were systematically searched using a combination of the following keywords: *Diet OR Dietary factors OR Dietary Intake OR intake OR Consumption OR food OR nutrition OR dietary protein OR antioxidant OR Nutrient OR Fibre OR carbohydrate OR fat OR fatty acid OR glycemic food OR vegetables OR Fruit OR vitamin OR caffeine OR fish OR alcohol OR calorie OR caloric OR Mediterranean AND Diabetic Retinopathy OR Diabetic Complications OR Microvascular Complications OR Diabetic Macular Edema*.

During preliminary searches, search keywords were initially based on similar reviews[24–27], which used broader generic dietary terms such as “diet OR dietary factors OR dietary intake OR consumption”. Furthermore, for an improved search comprehensiveness, additional specific dietary terms (such as “fibre” or “antioxidants”) based on areas our preliminary search found evidence of prior research, were included. This process continued until a search saturation point was found; i.e. the point at which additional terms showed no improvement in our search result. Relevant references identified from the bibliographies of pertinent articles or review papers were also retrieved.

### Study selection

Using our search strategy, 4265 titles were initially identified. Two authors (MW and RM) assessed the titles independently according to predefined inclusion criteria. Studies were then systematically excluded after detailed examination, if the title and abstract were not relevant. The full-text articles of studies deemed potentially relevant were also obtained, particularly if there was insufficient information within the abstract for exclusion.

#### Inclusion criteria

Our eligibility criteria were based on the PICOS (participants, intervention, comparability, outcomes, study design) framework recommended by the PRISMA guidelines[28].

**Fig 1:**
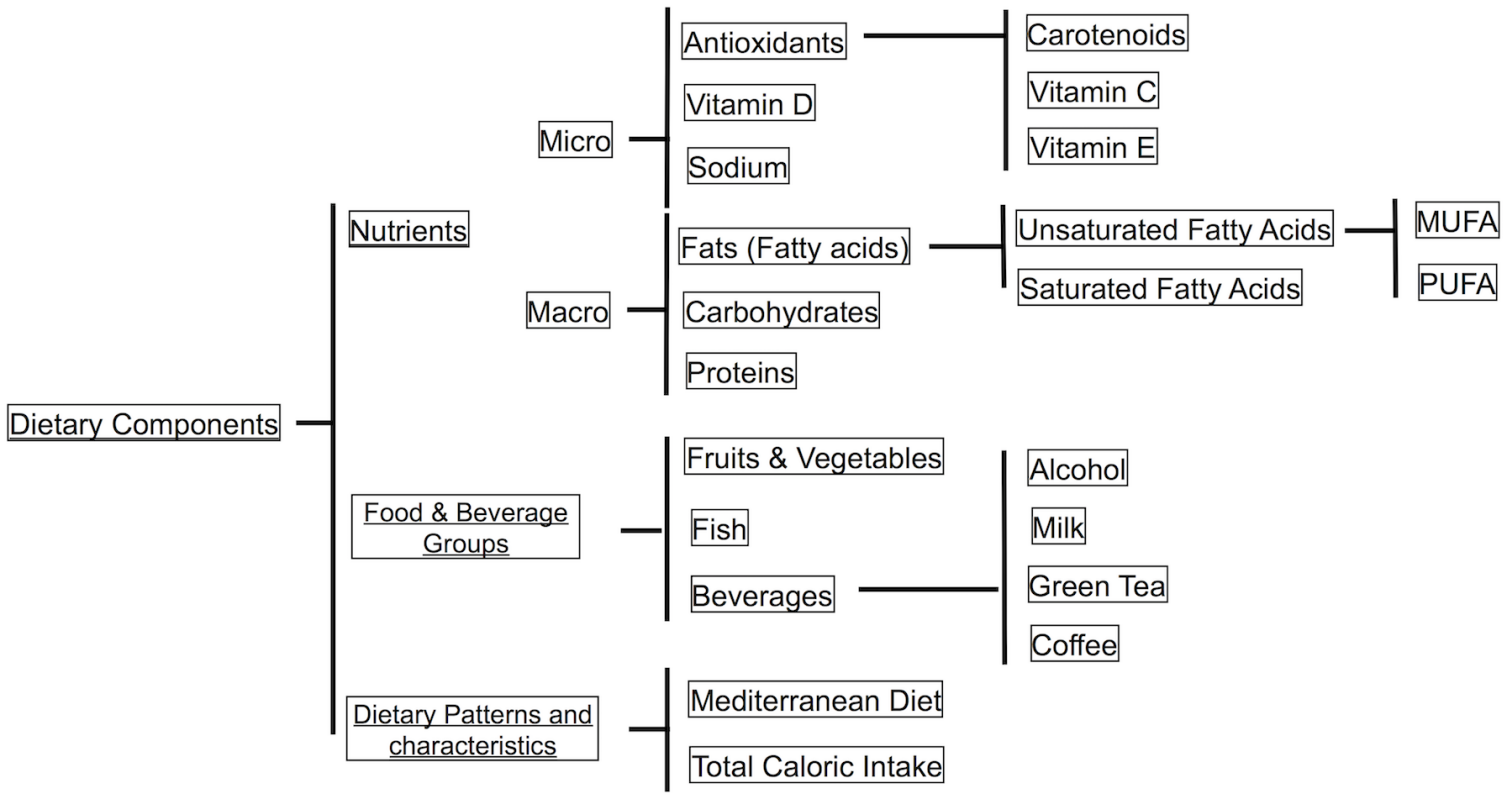
Overview of dietary components assessed in the studies included in our systematic review.

1. **Study Type**. We included both interventional (randomized controlled trials, and post-hoc analyses of interventional studies) and observational studies (cross-sectional, case-control and prospective).
2. **Participants**. Studies involved on human participants with type 1 diabetes, type 2 diabetes, or both.
3. **Exposures (interventions)**. Exposures had to measure a form of dietary intake, either through standard dietary methods (validated food frequency questionnaire, 24 hr- dietary recall, dietary history etc.), general interviewer-administered questionnaires or estimations from biomarker levels (such studies had to use biomarkers as a means to estimate dietary intake levels; before using these final estimated dietary intake levels as the main exposure). Dietary intake included the consumption of specific foods and beverages, the intake of micro/macronutrients, and adherence to meal patterns (Fig 1). Selected studies also had to specify how dietary intake was measured and quantified.
4. **Outcomes**. Outcomes were the prevalence, incidence or progression of DR or diabetic macula edema (DME). We accepted studies using different DR assessment methods, including but not limited to: fundus photography with or without mydriatic eyedrops; fundoscopy; direct or indirect ophthalmoscopy; and fluorescein angiography. We also included studies using different scales to grade DR severity, including but not limited to the Early Treatment Diabetic Retinopathy Study (ETDRS) scale[29] or the International Classification system of DR[30].

#### Exclusion criteria

The following types of papers were excluded:

1. Reviews
2. Studies on animals, and in-vitro / in-vivo studies.
3. Studies on a non-diabetic population, including participants with impaired glucose tolerance (IGT) or pre-diabetes.
4. Studies not defining the exposure or outcome variables
5. Studies measuring biomarkers in serum, blood, or urine without any link to dietary intake.
6. Exposures which involved multi-formulaic supplements (supplements which comprise of multiple different types of nutrients)
7. Studies only measuring outcomes of “retinal changes”, “visual acuity” or “microvascular complications” without specific reference to DR/DME.
8. Articles with insufficient data to draw conclusions. This included any form of data insufficiency which did not enable us to draw conclusions from/evaluate the study, (e.g. lack of exposure/outcome definitions, or lack of statistical analysis)

### Data extraction

A standardized data extraction form based on the “Strengthening the Reporting of Observational Studies in Epidemiology” (STROBE) statement[31] was used to extract the following relevant data from each included article: authors, year, study design, sample size, population characteristics, age of participants, dietary components, method of dietary assessment, DR outcome type, method of DR diagnosis, DR categorization, adjustment for confounders used in analysis, statistical methods used, and summary of key findings. Data extraction was done by one author (MW) and vetted by another (RM). Any potential disagreements were resolved through consulting the corresponding author (EL).

### Study quality evaluation

The quality of observational studies was assessed using a modified version of the Newcastle Ottawa Scale (NOS), a validated tool for evaluating observational study designs[32]. Originally designed to assess prospective and case-control studies, an adapted version of the NOS was used in the current study for the assessment of cross-sectional studies[33, 34]. The NOS uses three main bias-reducing criteria to award up to a maximum of 9 stars: (a) the selection and representativeness of the participants (maximum of 4 stars), (b) the comparability of groups (maximum of 2 stars), and (c) the ascertainment of exposure (for case-control) or outcome (for prospective and cross-sectional) (maximum of 3 stars). We also gave studies an additional star if they assessed dietary intake using validated dietary measurement tools, such as validated FFQs, or 24hr dietary record by dietician interviews, or if they estimated dietary intake from biomarker levels. Following previous reviews, studies assigned 0–4, 5–7, and ≥8 stars were considered as low, medium and high quality respectively[35–37].

For the evaluation of interventional studies (RCT), the Cochrane Collaboration Risk of Bias Tool was used, which measures risk of bias through seven criteria; sequence generation, allocation concealment, blinding of participants, masking of outcome assessment, incomplete outcome data, selective outcome reporting, and other sources of bias. Each criterion is individually graded according to whether it is deemed to have a high, low or unclear risk of bias. Studies which had a low risk of bias for all key domains were considered to be at an overall low risk of bias, studies with low or unclear risk of bias for all key domains were considered to be at a medium risk of bias, and studies with high risk of bias for one or more key domains were considered at an overall high risk of bias[38].

## Results

### Description of studies

**Fig 2:**
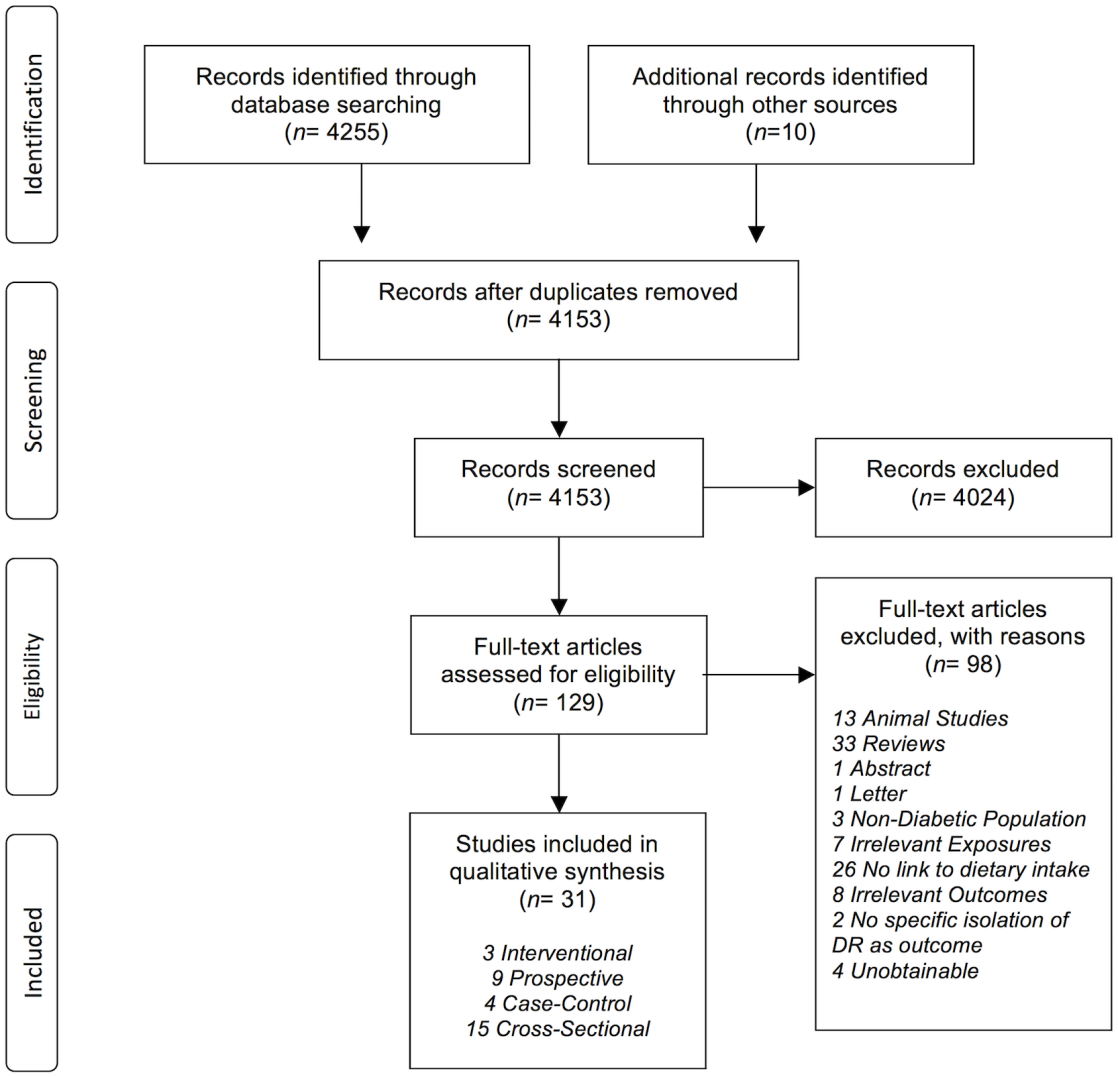
PRISMA Flow Diagram: Selection of included studies.

Of 4266 titles screened, 129 abstracts were extracted for detailed evaluation, of which 31 papers adhered to our inclusion criteria (Fig 2). They comprised of 3 interventional (RCTs) and 28 observational studies (9 prospective, 4 case-control, and 15 cross-sectional)

### Measurement of exposures and outcomes

For measurement of dietary intake in observational studies, most studies (n = 17) used standard dietary methods, including 24-hour recall (n = 2), food frequency questionnaires-FFQ (n = 14)[9–12, 15, 16, 18, 39–45] or 3-D food records (n = 1). Some studies used a general interviewer-administered questionnaire (n = 10)[17, 46–54], while one study estimated dietary sodium intake from urinary excretion levels. Studies determined DR outcomes through fundus photographs (n = 18), ophthalmologist examination (n = 6), direct ophthalmoscopy (n = 2) or linkage to patient’s previous medical/clinical/hospital records (n = 2) (Table 1).

**Table 1:** Characteristics of studies (n = 31).

Author, year	Sample Size	Diabetes	Age	Dietary Component	Dietary Assessment	DR outcome	Method of Diagnosing DR	DR Classification	Quality
**Interventional Studies (n = 3)**									
Houtsmuller, 1979	96	Any Diabetes	n.a.	Saturated Fat vs Unsaturated Fat	n.a.	Progression & incidence	Fundus Photography	None, NPDR, PDR, PRP	**High Bias**
Howard-Williams, 1985	149	Any diabetes	<66	Saturated Fat vs Unsaturated Fat	n.a.	Incidence	Ophthalmologist Examination	None, Retinopathy	**High Bias**
Diaz-Lopez, 2015	3614	T2DM	55–80	Med Diet	n.a.	Incidence	Ophthalmologist Examination	None, NPDR, PDR	**Moderate Bias**
**Prospective Studies (n = 9)**									
Young, 1984	296	Any Diabetes	20–59	Alcohol	Self Report in general questionnaire	Incidence	Direct Ophthalmoscopy	Modified ETDRS	**8**
Moss, 1993	Young: 439 Older: 478	Any Diabetes	21–94	Alcohol	Self Report in general questionnaire	Incidence & progression	Fundus Photography	Modified ETDRS	**9**
Roy, 2010	469	T1DM	NR*	MUFA, PUFA, Oleic Acid, Protein, Dietary Fibre, carbohydrates, sodium, high caloric	Validated FFQ	Progression & Incidence	Fundus Photography	Modified ETDRS	**9**
Cundiff, 2005	1412	T1DM	13–39	MUFA, PUFA, Carbohydrates, Protein, Dietary Fibre, Sodium, Alcohol, High Calories	Dietary History Interview	Progression	Fundus Photography	Modified ETDRS	**8**
Lee, 2010	1239	T2DM	55–81	Alcohol	Self Report in general questionnaire	Progression	Fundus Photography	Modified ETDRS	**9**
Tanaka, 2013	978	T2DM	40–70	Fruit & vegetables, Vitamin C, Vitamin E, Carotenoids	Validated FFQ + 24 Hr Dietary Recall	Incidence	Ophthalmologist Examination	International Classification System	**10**
Horikawa, 2014	978	T2DM	40–70	Sodium	Validated FFQ	Progression& incidence	Ophthalmologist Examination	International Classification System	**10**
Horikawa, 2017	978	T2DM	40–70	Carbohydrates	Validated FFQ	Progression & Incidence	Ophthalmologist Examination	International Classification System	**10**
Sala-Vila, 2016	3482	T2DM	55–80	PUFA (LCw3) & Oily Fish	Validated FFQ	Incidence	Clinical and Hospital Records	None, NPDR, PDR	**9**
**Case-Control Studies (n = 4)**									
Giuffre, 2004	Cse = 45 Ctr: 87	Any Diabetes	>40	Alcohol	Self Report in general questionnaire	Prevalence	Direct Opthalmoscopy + Fundus Photography	ETDRS	**7**
Ma, 2014	Cse: 100 Ctr: 100	T2DM	>18	Green Tea	Questionnaire on tea consumption	Prevalence	Fundus Photography	ETDRS	**8**
Alcubierre, 2015	Cse: 139 Ctr: 144	T2DM	NR	Vitamin D, calcium	Validated FFQ	Prevalence	Ophthalmologist Examination	International Classification System	**8**
Alcubierre, 2016	Cse: 146 Ctr: 148	T2DM	40–75	MUFA, PUFA, Oleic Acid, Carbohydrates, Protein, Dietary Fibre,	Validated FFQ	Prevalence	Ophthalmologist Examination	International Classification System	**10**
**Cross-Sectional Studies (n = 15)**									
Roy, 1989	34	Any Diabetes	NR	MUFA, PUFA, Carbohydrates, Protein, Dietary Fibre	3-d Food Record	Prevalence	Fundus Photography	Modified Airlie House Classification	**5**
Moss, 1992	Young: 891 Older: 987	Any Diabetes	2–96	Alcohol	Self Report in general questionnaire	Prevalence	Fundus Photography	Modified Airlie House	**9**
Mayer-Davis, 1998	387	T2DM	20–74	Vitamin C, E & Beta-Carotene	24 Hr Dietary Recall	Prevalence	Dilated Fundus Photography	Modified Airlie House Criteria	**9**
Millen, 2004	1353	Any Diabetes	45–65	Vitamin C & E	Validated FFQ	Prevalence	Non-Dilated Fundus Photography	Modified Airlie House	**8**
Beulens, 2008	1857	T1DM	15–60	Alcohol	Self Report in general questionnaire	Prevalence	Dilated Fundus Photography	None, background, proliferative	**10**
Ganesan, 2012	1261	Any Diabetes	>40	Dietary Fibre	Validated Fibre Questionnaire	Prevalence	Dilated Fundus Photography	Modified ETDRS	**10**
Harjutsalo, 2013	3608	T1DM	NR	Alcohol	Self Report in general questionnaire	Prevalence	History of Laser Photocoagulation	Severe DR Vs None	**8**
Lugo-Radillo, 2013	88	Any Diabetes	NR	Fruit & Vegetables	Oral Questionnaire on F&V Consumption	Prevalence	Ophthalmologist Examination	International Classification System	**5**
Mahoney, 2014	155	Any Diabetes	>40	Fruit & Vegetables	Validated FFQ	Prevalence	Undilated Fundus Photography	ETDRS	**8**
Engelen, 2014	1880	T1DM	15–60	Sodium	Estimated from Urinary Sodium Excretion	Prevalence	Fundus Photography	None, NPDR, PDR	**7**
Kumari, 2014	353	Any Diabetes	21–95	Coffee	Questionnaire on coffee consumption	Prevalence	Dilated Fundus Photography	Modified Airlie House Classification	**8**
Sasaki, 2015	379	Any Diabetes	>18	Vitamin C, E, B-Carotene, MUFA, PUFA, carbohydrates, protein	Validated FFQ	Prevalence	Fundus Photography	Modified ETDRS	**8**
Fenwick, 2015	395	T2DM	>18	Alcohol	Validated FFQ	Prevalence	Undilated Fundus Photography	ETDRS	**10**
Millen, 2016	1305	Any Diabetes	45–65	Vitamin D, Fish, Milk	Validated FFQ	Prevalence	Fundus Photography	Modified Airlie House	**9**
Sahli, 2016	1430	Any Diabetes	45–65	Carotenoids (Lutein)	Validated FFQ	Prevalence	Non-Dilated Fundus Photography	ETDRS	**9**

### Methodological quality

Of 28 observational studies, the majority had high NOS scores, with 25 classified as “high quality” (>8 stars) and 3 classified as “moderate quality” (5–7 stars). Of the 3 interventional studies, 2 and 1 had a high-risk and medium risk of bias, respectively (Table 1).

### Associations between micronutrient intake and DR

#### Antioxidants

Carotenoids, Vitamin C and Vitamin E are common antioxidants, and their associations with DR are reflected in Table 2.

**Table 2:** Dietary intake of micro-nutrients and DR.

Author, year	Association	Study Design	Quality	Dietary Factor	Sample Size	DR outcome type	Confounders adjusted for	Statistical methods	Main Findings
**Antioxidants**									
**Carotenoids**									
Tanaka, 2013	Protective	Prospective	10	Carotenoids	978	Incidence	Age, sex, BMI, HbA1c, duration of diabetes, treatment by insulin, treatment by oral hypoglycemic agents without insulin, systolic blood pressure, LDL Cholesterol, HDL cholesterol, triglycerides, smoking, alcohol, physical activity, total energy intake, proportions of dietary protein, fat, carbohydrate, saturated fatty acids, n-6 PUGA and n-3 PUFA, cholesterol & sodium	Multivariate Cox Regression	Highest Intake Quartile (Q4) vs lowest Intake Quartile (Q1), HR: 0.52 (0.33–0.81)
Mayer-Davis, 1998	NS	Cross Sectional	9	Carotenoids (B-Carotene)	387	Prevalence	Age, duration of diabetes, ethnicity, glycosylated hemoglobin, hypertension, caloric intake, gender & insulin use.	Multivariable logistic regression	No significant associations with DR (Data not reported)
Sahli, 2016	NS	Cross Sectional	9	Carotenoids (Lutein)	1430	Prevalence	HbA1c, blood pressure, duration of diabetes, race, total energy consumption & study center	Multivariable logistic regression	Intake Q3 vs Q1, OR: 1.54 (0.96–2.47) Intake Q4 vs Q1, OR: 1.41 (0.87–2.28)
Sasaki, 2015	NS	Cross Sectional	8	Carotenoids (B-Carotene)	379	Prevalence	Energy Intake	Data not reported	No significant associations with DR (Data not reported)
**Vitamin C**									
Tanaka, 2013	Protective	Prospective	10	Vitamin C	978	Incidence	Age, sex, BMI, HbA1c, duration of diabetes, treatment by insulin, treatment by oral hypoglycemic agents without insulin, systolic blood pressure, LDL Cholesterol, HDL cholesterol, triglycerides, smoking, alcohol, physical activity, total energy intake, proportions of dietary protein, fat, carbohydrate, saturated fatty acids, n-6 PUGA and n-3 PUFA, cholesterol & sodium	Multivariate Cox Regression	Intake Q4 vs Q1, HR: 0.61 (0.39–0.96)
Mayer-Davis, 1998	Risk	Cross Sectional	9	Vitamin C	387	Prevalence	Age, duration of diabetes, ethnicity, glycosylated hemoglobin, hypertension, caloric intake, gender & insulin use.	Multivariable logistic regression	Intake 9^th^ Decile vs 1^st^ Quintile, OR: 2.21 (p = 0.011)
Millen, 2004	NS	Cross Sectional	8	Vitamin C	1353	Prevalence	Total energy intake, race, duration of diabetes, serum glucose, hypertension, BMI, waist-hip ratio, smoking, alcohol, drinking status, plasma triacylglycerol, plasma cholesterol, hematocrit value, prevalent coronary heart disease, diabetes treatment group, &use of oral hypoglycemic agents or use of insulin	Multivariable logistic regression	Intake Q4 vs Q1, OR: 1.4 (0.8–2.4)
Sasaki, 2015	NS	Cross Sectional	8	Vitamin C	379	Prevalence	Energy Intake	Data not reported	No significant associations with DR (Data not reported)
**Vitamin E**									
Mayer-Davis, 1998	Risk (in insulin non-taking subjects)	Cross Sectional	9	Vitamin E	387	Prevalence	Age, duration of diabetes, ethnicity, glycosylated hemoglobin, hypertension, caloric intake, gender & insulin use.	Multivariable logistic regression	Insulin Subjects: No Association Non-Insulin taking Subjects: Intake 10^th^ Decile vs 1^st^ Quintile, OR: 3.79 (p<0.02)
Tanaka, 2013	NS	Prospective	10	Vitamin E	978	Incidence	Age, sex, BMI, HbA1c, duration of diabetes, treatment by insulin, treatment by oral hypoglycemic agents without insulin, systolic blood pressure, LDL Cholesterol, HDL cholesterol, triglycerides, smoking, alcohol, physical activity, total energy intake, proportions of dietary protein, fat, carbohydrate, saturated fatty acids, n-6 PUGA and n-3 PUFA, cholesterol & sodium	Multivariate Cox Regression	Intake Q4 vs Q1, HR: 0.84 (0.51–1,40)
Millen, 2004	NS	Cross Sectional	8	Vitamin E	1353	Prevalence	Total energy intake, race, duration of diabetes, serum glucose, hypertension, BMI, waist-hip ratio, smoking, alcohol, drinking status, plasma triacylglycerol, plasma cholesterol, hematocrit value, prevalent coronary heart disease, diabetes treatment group & use of oral hypoglycemic agents or use of insulin	Multivariable logistic regression	Intake Q4 vs Q1, OR: 1.4 (0.8–2.3)
Sasaki, 2015	NS	Cross Sectional	8	Vitamin E	379	Prevalence	Energy Intake	Data not reported	No significant associations with DR (Data not reported)
**Vitamin D**									
Millen, 2016	NS	Cross-Sectional	9	Vitamin D	1305	Prevalence	Race, duration of diabetes, HbA1c & hypertension	Multivariate Logistic Regression	Intake Q4 Vs Q1, OR: 1.20 (0.76–1.89)
Alcubierre, 2015	NS	Case-Control	8	Vitamin D	Case: 139 Ctrl: 144	Prevalence	NIL	Chi-Squared	No significant associations with DR (p = 0.93)
**Calcium**									
Alcubierre, 2015	NS	Case-Control	8	Calcium	Case: 139 Ctrl: 144	Prevalence	NIL	Chi-Squared	No significant associations with DR (p = 0.65)
**Sodium**									
Roy, 2010	Risk (For DME) NS for DR	Prospective	10	Sodium	469	Progression & Incidence	Total caloric intake, age, sex, physical exercise, glycated hemoglobin, oleic acid intake, protein intake, carbohydrate intake & hypertension	Multivariable Logistic Regression	No significant associations with DR For DME, Intake Q4 Vs Q1, OR: 1.43 (1.10–1.86)
Horikawa, 2014	NS	Prospective	10	Sodium	978	Progression& incidence	Age, Sex, BMI, HbA1c, diabtes duration, LDL cholesterol, HDL cholesterol, log-transformed triglycerides, insulin treatment, treatment by lipid-lowering agents, current smoking, alcohol intake, energy intake, sodium intake & physical activity	Multivariable Cox Regression	Intake Q4 Vs Q1, HR: 1.10 (0.75–1.61)
Cundiff, 2005	NS	Prospective	8	Sodium	1412	Progression	Energy Intake	Spearman Correlation	Sodium in mg/kcal against DR progression rate, r = 0.02 (p = 0.47)
Engelen, 2014	NS	Cross-Sectional	7	Sodium	1880	Prevalence	Age, sex, BMI, smoking, urinary potassium excretion, antihypertensive medication, total energy intake, physical activity, sat fat intake, protein intake, fibre intake & alcohol intake	Multivariable Logistic Regression	Per 1g/day increase in dietary salt intake, OR: 1.00 (0.96–1.04)

Carotenoids—Using a prospective design, Tanaka and associates[15] found carotenoids to be protective of incident DR using a multivariate cox regression analysis (4^th^ (highest) intake quartile [Q4] vs. 1^st^ (lowest) intake quartile [Q1], Hazard Ratio [HR]: 0.52, 95% confident interval [CI]: 0.33–0.81). On the other hand, the other three cross-sectional studies reported non-significant associations between carotenoids and DR[12, 44, 55].

Vitamin C—Similarly, Tanaka and associates[15] reported a protective relationship between Vitamin C intake and incident DR (Q4 vs. Q1, HR, 95% CI: 0.61, 0.39–0.96), in contrast to a cross-sectional study by Mayer-Davis and colleagues[55] that reported a risk association between vitamin C intake and prevalent DR (9^th^ decile vs. 1^st^ quintile, Odds Ratio [OR]: 2.21, p = 0.01). The remaining two other cross-sectional studies found non-significant relationships between Vitamin C intake and DR[11, 12].

Vitamin E*—*Mayer-Davis and colleagues’ cross-sectional investigation[55] found a risk association between Vitamin E and prevalent DR (10^th^ decile vs 1^st^ quintile, OR: 3.79, p<0.002), but only within non-insulin taking patients. All remaining studies (two prospective, one cross-sectional) reported no significant associations. [11, 12, 15].

Overall, the associations between these common antioxidants and DR remain inconsistent.

#### Vitamin D

The only two studies[9, 18] that examined the association between dietary vitamin D intake and DR did not find any significant associations. (Table 2).

#### Sodium

The evidence overwhelmingly suggests (n = 4) that sodium intake is not associated with DR[10, 13, 42, 56] (Table 2). However, one study reported a risk association between sodium intake and DME progression[10] (Q4 vs. Q1, OR, 95% CI: 1.43, 1.10–1.86).

### Associations between macronutrient intake and DR

#### Mono-Unsaturated Fatty Acids (MUFA)

Alcubierre and associates,[39] using a case-control design, reported a protective association between MUFA intake and DR (high MUFA intake vs. low MUFA intake, OR, 95% CI: 0.42, 0.18–0.97). In contrast, a prospective study by Cundiff and colleagues[13] reported a risk relationship between MUFA intake and DR progression, but did not adjust for important confounders such as duration of diabetes, HbA1c or diabetes treatment. The remaining majority of studies (two cross-sectional and one prospective) found no significant relationships between MUFA intake and DR[10, 12, 14](Table 3). Two studies that further analyzed the effects of Oleic acid (a specific MUFA) on DR also reported contrasting results[10, 39].

**Table 3:** Dietary intake of macro-nutrients and DR.

Author, year	Association	Study Design	Quality	Dietary Factor	Sample Size	DR outcome type	Confounders adjusted for	Statistical methods	Main Findings	
**Dietary Fats / lipids**										
**Mono-Unsaturated Fatty Acids (MUFA)**										
Alcubierre, 2016	Protective	Case-Control	10	MUFA	Case: 146 Ctrl: 148	Prevalence	Age, gender, diabetes duration, energy intake, educational level, physical activity, waist circumference, systolic BP, HDL cholesterol & diabetes treatment	Multivariable Logistic Regression	High MUFA consumption vs Low MUFA consumption, OR: 0.42 (0.18–0.97)	
Cundiff, 2005	Risk	Prospective	8	MUFA	1412	Progression	Energy Intake	Spearman Correlation	MUFA in %/kcal against DR progression rate, r = 0.12 (p = 0.001)	
Roy, 2010	NS	Prospective	9	MUFA	469	Progression & Incidence	Total caloric intake, total fat, sat fat, oleic acid, linoleic acid, protein, fiber, cholesterol & sodium intakes	Multivariable Logistic Regression	No significant associations with DR (Data not reported)	
Sasaki, 2015	NS	Cross Sectional	10	MUFA	379	Prevalence	Age, gender, HBA1C, mean arterial pressure & diabetes duration	Multivariable logistic regression models	Per 10 energy-adjusted g/d increase, OR: 1.19 (0.74–1.92)	
Roy, 1989	NS	Cross-Sectional	5	MUFA	34	Prevalence	Energy Intake	t-test	No significant associations with DR (Data not reported)	
**Poly-Unsaturated Fatty Acids (PUFA)**										
Sala-Vila, 2016	Protective	Prospective	9	PUFA (LCw3)	3482	Incidence	Age, gender, BMI, intervention group, yeasr after diagnosis of diabetes, use of insulin, use of oral hypoglycemic agents, smoking, systolic BP, hypertension, physical activity, adherence to meddiet.	Cox Proportional Hazard Model	>500mg/d Vs <500mg/d, HR: 0.52 (0.31–0.88)	
Sasaki, 2015	Protective for well controlled diabetics	Cross Sectional	10	PUFA	379	Prevalence	Age, gender, HBA1C, mean arterial pressure & diabetes duration	Multivariable logistic regression models	All subjects: Per 10 energy-adjusted g/d increase, OR: 0.67 (0.37–1.20) Well controlled Diabetics: Per 10 energy-adjusted g/d increase, OR: 0.18 (0.06–0.59)	
Cundiff, 2005	Risk	Prospective	8	PUFA	1412	Progression	Energy Intake	Spearman Correlation	PUFA in %/kcal against DR progression rate, r = 0.09 (r = 0.004)	
Roy, 2010	NS	Prospective	9	PUFA	469	Progression & Incidence	Total caloric intake, total fat, sat fat, oleic acid, linoleic acid, protein, fiber, cholesterol & sodium intakes	Multivariable Logistic Regression	No significant associations with DR (Data not reported)	
Alcubierre, 2016	NS	Case-Control	10	PUFA	Case: 146 Ctrl: 148	Prevalence	Age, gender, diabetes duration, energy intake, educational level, physical activity, waist circumference, systolic BP, HDL cholesterol & diabetes treatment	Multivariable Logistic Regression	High PUFA consumption vs Low MUFA consumption, OR: 0.99 (0.69–1.41)	
Roy, 1989	NS	Cross-Sectional	5	PUFA	34	Prevalence	Energy Intake	t-test	No significant associations with DR (Data not reported)	
**Oleic Acid**										
Alcubierre, 2016	Protective	Case-Control	10	Oleic Acid	Case: 146 Ctrl: 148	Prevalence	Age, gender, diabetes duration, energy intake, educational level, physical activity, waist circumference, systolic BP, HDL cholesterol & diabetes treatment	Multivariable Logistic Regression	High Intake Tertile (T3) vs Lowest Intake Tertile (T1), OR: 0.37 (0.16–0.85)	
Roy, 2010	NS	Prospective	9	Oleic Acid	469	Progression & Incidence	Total caloric intake, total fat, sat fat, oleic acid, linoleic acid, protein, fiber, cholesterol & sodium intakes	Multivariable Logistic Regression	No Significant associations with DR (Data not reported)	
**Interventional Studies**										
Houtsmuller, 1979	Protective	Interventional	High Bias	Unsaturated Fats	96	Progression	Matched for gender	Saturated Fat Diet Vs Unsaturated Fat Diet Males (n = 52, 26 on each diet) P<0.001 Females (n = 44, 22 on each diet) P<0.025		
Howard-williams, 1985	NS	Interventional	High Bias	PUFA	149	Incidence	Matched for age, sex & BMI	Persons on modified fat diet (PUFA: saturated fat ratio, 0.3) vs persons on low carb diet (PUFA: Saturated fat ratio, 0.9) All patients (n = 149) No difference between two groups (chi-squared, p = 0.69) Dietary compliers (n = 58) No difference between two groups (chi-squared, p = 0.13)		
Carbohydrates										
Cundiff, 2005	Protective	Prospective	8	Carbohydrates	1412	Progression	Energy Intake	Spearman Correlation		Carbohydrates in %/kcal against DR progression rate, r = -0.11 (p<0.001)
Roy, 1989	Protective	Cross-Sectional	5	Carbohydrates	34	Prevalence	Energy Intake	t-test		Persons without retinopathy vs Persons with retinopathy (p<0.05)
Horikawa, 2017	NS	Prospective	10	Carbohydrates	978	Incidence and Progression	Age, sex, BMI, HbA1C, Diabetes Duration, systolic BP, LDL-cholesterol, HDL-cholesterol, triglycerides, treatment by insulin, treatment by antihypertensive agents, treatment by lipid-lowering agents, current smoker, alcohol intake, energy intake & physical activity	Multivariable Cox Regression Models		Highest Intake Tertile (T3) vs lowest Intake Tertile (T1), HR: 1.00 (0.72–1.38)
Roy, 2010	NS	Prospective	9	Carbohydrates	469	Progression & Incidence	Total caloric intake, total fat, sat fat, oleic acid, linoleic acid, protein, fiber, cholesterol & sodium intakes	Multivariable Logistic Regression		No significant associations with DR (Data not reported)
Alcubierre, 2016	NS	Case-Control	10	Carbohydrates	Case: 146 Ctrl: 148	Prevalence	Age, gender, diabetes duration, energy intake, educational level, physical activity, waist circumference, systolic BP, HDL cholesterol & diabetes treatment	Multivariable Logistic Regression		High Intake Tertile (T3) vs lowest intake tertile (T1), OR: 1.18 (0.45–3.09)
Sasaki, 2015	NS	Cross Sectional	8	Carbohydrates	379	Prevalence	Energy Intake	Chi-Squared		No significant associations with DR (data not reported)
Protein										
Cundiff, 2005	Protective	Prospective	8	Protein	1412	Progression	Energy Intake	Spearman Correlation		Protein in %/kcal against DR progression rate, r = -0.6 (p = 0.0188)
Roy, 1989	Risk	Cross-Sectional	5	Protein	34	Prevalence	Energy Intake	t-test		Persons without retinopathy vs Persons with retinopathy (p<0.02)
Roy, 2010	NS	Prospective	9	Protein	469	Progression & Incidence	Total caloric intake, total fat, sat fat, oleic acid, linoleic acid, protein, fiber, cholesterol & sodium intakes	Multivariable Logistic Regression		No Significant associations with DR (Data not reported)
Alcubierre, 2016	NS	Case-Control	10	Protein	Case: 146 Ctrl: 148	Prevalence	Age, gender, diabetes duration, energy intake, educational level, physical activity, waist circumference, systolic BP, HDL cholesterol & diabetes treatment	Multivariable Logistic Regression		Highest protein intake tertile (T3) vs lowest protein intake tertile (T1), OR: 1.24 (0.49–3.16)
Sasaki, 2015	NS	Cross Sectional	8	Protein	379	Prevalence	Energy Intake	Chi-Squared		No Significant associations with DR (Data not reported)

#### Poly-Unsaturated Fatty Acids (PUFA)

A prospective study by Sala-Vila and associates[45] found those adhering to the dietary long-chain omega-3 PUFA (LCω3PUFA) recommendation of at least 500mg/day to be at lower risk of incident DR than those who did not adhere (HR, 95% CI: 0.52, 0.31–0.99). Similarly, though Sasaki and colleagues[12] found no overall association between PUFA intake and DR, they reported a protective asociation within patients with well-controlled diabetes (OR, 95% CI: 0.18, 0.06–0.59). In contrast to these two studies, Cundiff and colleagues[13] reported a risk association between a larger percentage of caloric intake as PUFA’s and DR progression, though again this result was not adjusted for DR confounders. The remaining three other studies[10, 14, 39] reported no significant relationships between PUFA intake and DR.

Interventional studies have been equally equivocal; a 1979 study by Houtsmuller and associates[57] reported a significant reduction in DR progression among participants on an unsaturated fat diet rich in linoleic acid, compared to those on a saturated fat diet. In contrast, a later study by Howard-Williams and colleagues[58] reported no significant differences in incident DR between compliers of a modified fat diet (high PUFA to saturated fat ratio) and those on a low carbohydrate diet (low PUFA to saturated fat ratio).

#### Carbohydrates

Two studies (one cross-sectional, one prospective) found carbohydrate intake to be protective for DR[13, 14] (Table 3), but neither study adjusted for relevant confounders. In contrast, the remaining four studies[10, 12, 39, 43]—three of which used fully adjusted multivariable models—reported non-significant relationships between carbohydrate intake and DR.

#### Protein

A prospective study by Cundiff and colleagues reported those with a larger percentage of caloric intake as proteins to be at lower risk of DR progression. In contrast, a cross-sectional study by Roy and associates reported a risk association between protein intake and prevalent DR[13, 14]. However, both studies did not adjust for relevant confounders. The remaining three studies[10, 12, 39] that adjusted for confounders reported non-significant relationships between dietary protein intake and DR (Table 3).

### Associations between food and beverage intake and DR

#### Fruits, vegetables and dietary fibre

Two studies (one prospective, one cross-sectional) reported a protective association between the intake of fruits and vegetables and DR, in contrast to one cross-sectional study that reported non-significant associations. Similarly, for dietary fibre, the majority of studies(two prospective, two cross-sectional) reported a protective effect of increased dietary fibre intake on DR[13–16, 41] (Table 4), in contrast to two other studies that reported non-significant associations.

**Table 4:** Dietary intake of foods, beverages, dietary patterns and DR.

Author, year	Association	Study Design	Quality	Dietary Factor	Sample size	DR outcome type	Confounders adjusted for	Statistical methods	Main Findings
**Dietary Fibre**									
Tanaka, 2013	Protective	Prospective	10	Fruits, Vegetables, & Dietary Fibre	978	Incidence	Age, sex, BMI, HBA1C, Duration of Diabetes, Treatment by insulin, treatment by oral hypoglycemic agents without insulin, systolic blood Pressure, LDL Cholesterol, HDL cholesterol, Triglycerides, smoking, alcohol, physical activity, total energy intake, proportions of dietary protein, fat, carbohydrate, saturated fatty acids, n-6 PUGA and n-3 PUFA, cholesterol & Sodium	Multivariate Cox Regression	Veg & Fruit intake Q4 vs Q1, HR: 0.59 (0.37–0.92) Fruit intake Q4 Vs Q1, HR: 0.48(0.32–0.71) Dietary Fibre intake Q4 Vs Q1, HR: 0.63 (0.38–1.03)
Cundiff, 2005	Protective	Prospective	8	Dietary Fibre	1412	Progression	Energy Intake	Spearman Correlation	Dietary fibre in g/1000kcal against DR progression rate, r = -0.10 (p = 0.002)
Ganesan, 2012	Protective	Cross Sectional	10	Dietary Fibre	1261	Prevalence	Age, Gender, duration of diabetes, BP, BMI, glycosylated hemoglobin, serum lipids, smoking status & SES.	Multivariable Logistic Regression	Low-fibre diet Vs High fibre diet for any DR, OR: 1.41 (1.02–1.94) Low fibre diet Vs High fibre diet for VTDR, OR: 2.24 (1.01–5.02)
Roy, 1989	Protective	Cross-Sectional	5	Dietary Fibre	34	Prevalence	Duration of diabetes	t-test	Persons without retinopathy vs Persons with retinopathy, (p<0.01)
Roy, 2010	NS	Prospective	9	Dietary Fibre	469	Progression & Incidence	Total caloric intake, total fat, sat fat, oleic acid, linoleic acid, protein, fiber, cholesterol & sodium intakes	Multivariable Logistic Regression	No significant associations with DR (Data not reported)
Alcubierre, 2016	NS	Case-Control	10	Dietary Fibre	Case: 146 Ctrl: 148	Prevalence	Age, gender, diabetes duration, energy intake, educational level, physical activity, waist circumference, systolic BP, HDL Cholesterol & Diabetes treatment	Multivariable Logistic Regression	Highest Fibre intake tertile (T3) vs lowest Fibre intake tertile (T1), OR: 0.76 (0.33–1.76)
Fruits & vegetables									
Mahoney, 2014	Protective	Cross Sectional	8	Fruit & Vegetables	155	Prevalence	Age, Gender, Ethnicity, BMI, HbA1C, Physical activity, diabetic medications, CVD, cancer, stroke, & homocysteine	Multivariable Logistic Regression	Per 10 Unit increase in HFVC (High-flavonoid Fruit and Vegetable consumption) Index, OR:0.67 (0.45–0.99) With adjustment for duration of diabetes (n = 115) per 10 unit increase in HFVC index, OR:0.59 (P = 0.03)
Lugo-Radillo, 2013	NS	Cross-Sectional	5	Fruit & Vegetables	88	Prevalence	NIL	Binary Logistic Regression	High fruit & vegetable diet vs low fruit & vegetable diet, OR: (OR = 1.2, 0.3–6.2) High fruit consumption vs Low fruit consumption, OR: 1.8 (0.4–8.9) High vegetable consumption vs Low vegetable consumption, OR: 0.9 (0.3–2.9)
**Fish**									
Sala-Vila, 2016	Protective	Prospective	9	"Oily Fish"	3482	Incidence	Age, gender, BMI, intervention group, year after diagnosis of diabetes, use of insulin, use of oral hypoglycemic agents, smoking, systolic BP, hypertension, physical activity & adherence to med diet.	Cox Proportional Hazard Model	>2 servings a week vs <2 servings a week, HR: 0.41 (0.23–0.72)
Millen, 2016	Protective	Cross-Sectional	9	Fish	1305	Prevalence	Race, duration of diabetes, HBA1C & Hypertension	Multivariate Logistic Regression	Dark fish >1 a week vs never, OR: 0.32 (0.14–0.78) Other fish >1 a week vs never, OR: 1.16 (0.70–1.92)
**Green Tea**									
Ma, 2014	Protective	Case-Control	8	Green Tea	Case:100 Ctrl: 100	Prevalence	Education, BMI, systolic BP, smoking, alcohol, duration of diabetes, insulin therapy, family history of diabetes, physical activity & fasting blood glucose	Multivariable logistic regression	Regular chinese green tea drinker vs non-regular chinese green tea drinker, OR: 0.48 (0.24–0.97)
**Coffee**									
Kumari, 2014	NS	Cross Sectional	9	Coffee	353	Prevalence	Age, gender, smoking, BMI, HbA1c, creatinine, education level, duration of diabetes, family history of diabetes, history of hypertension, ischemic heart disease, stroke, dyslipidemia & cancer	Multivariable logistic regression	Coffee drinker vs never/rarely, OR: 1.36 (0.69–2.69)
**Milk**									
Millen, 2016	NS	Cross-Sectional	9	Milk	1305	Prevalence	Race, duration of diabetes, HBA1C & Hypertension	Multivariate Logistic Regression	Skim Milk, OR: 1.13 (0.67–1.91) Whole Milk, OR: 0.88 (0.35–2.23)
Alcohol									
Beulens, 2008	Protective	Cross-Sectional	10	Alcohol	1857	Prevalence	Age, gender, centre, smoking, physical activity, duration of diabetes, systolic BP, BMI, presence of CVD and HbA1C	Multivariable Logistic Regression	Mod Vs Abstain, OR: 0.60 (0.37–0.99)
Fenwick, 2015	Protective	Cross-Sectional Study	10	Alcohol	395	Prevalence	Age, Gender, Poor Diabetes Control, Diabetes Duration, Smoking BMI, SBP, insulin use and presence of at least one other diabetic Complication	Multivariable Logistic Regression	Mod Vs Abstain, OR: 0.47 (0.26–0.95) Mod White Wine Vs Abstain, OR:0.48 (0.25–0.91) Mod Fortified Wine Vs Abstain, OR: 0.15 (0.04–0.62)
Moss, 1992	Protective	Cross Sectional	9	Alcohol	Younger: 891 Older:987	Prevalence	Duration of diabetes, age, glycosylated hemoglobin, diastolic BP, use of insulin	Multivariable Logistic Regression	Younger onset diabetics Per 1oz/day increase in alcohol consumption for PDR, OR: 0.49, (0.27–0.92) Older onset: no significant associations
Harjutsalo, 2013	Protective	Cross-Sectional	8	Alcohol	3608	Prevalence	Age at onset of diabetes, sex, duration of diabetes, triglycerides, HDL cholesterol, HbA1C, social class, BMI, smoking status, hypertension and lipid-lowering medication	Multivariable Logistic Regression	Abstain Vs Light, OR: 1.42 (1.11–1.82) Former Use Vs Light, OR: 1.73 (1.07–2.79)
Young, 1984	Risk	Prospective	8	Alcohol	296	Incidence	Duration of diabetes, glycemic control & impotence	Multivariable Logistic Regression	Heavy consumption Vs None-Mod consumption, RR: 2.25 (1.15–4.42)
Cundiff, 2005	NS	Prospective	8	Alcohol	1412	Progression	Energy Intake	Spearman Correlation	No Significant association with DR (p = 0.26)
Lee, 2010	NS	Prospective	9	Alcohol	1239	Progression	Age, Gender, Smoking, BMI, HbA1C, Systolic BP, duration of diabetes and ethnicity	Multivariable Logistic Regression	Mod Vs None, OR: 1.08 (0.70–1.67) Heavy Vs None, OR: 1.07 (0.54–2.13)
Moss, 1993	NS	Prospective	9	Alcohol	Younger: 439 Older:478	Incidence & progression	Glycosylated Hemoglobin, Age, Sex	Multivariable Logistic Regression	Younger onset diabetics Per 1oz/day increase in alcohol consumption on DR incidence, OR: 2.09 (0.04–1.07) Per 1oz/day increase in alcohol consumption on DR progression, OR: 1.25 (0.75–2.08) Older onset diabetics Per 1oz/day increase in alcohol consumption on DR incidence, OR: 0.75 (0.4–1.42) Per 1oz/day increase in alcohol consumption on DR progression, OR: 0.73 (0.4–1.20)
Giuffre, 2004	NS	Case-Control	7	Alcohol	Case: 45 Ctrl: 87	Prevalence	Duration of Diabetes, Duration of Treatment with oral drugs, Duration of insulin treatment	Multivariable Logistic Regression	No Significant Association with DR (Data not reported)
**Mediterranean Diet**									
Diaz-Lopez, 2015	Protective	Interventional	Moderate Bias	Med Diet	3614	Incidence of DR	Age, sex, BMI, Waist circumference, Smoking, physical activity, educational level, hypertension, dyslipidemia, family history of premature coronary heart disease, and baseline adherence.	Multivariate Cox Regression	Med Diet vs Control Diet, HR: 0.60 (0.37–0.96)
**Caloric Intake**									
Roy, 2010	Risk	Prospective	10	Caloric Intake	469	Progression & Incidence	Total caloric intake, age, sex, physical exercise, glycated hemoglobin, oleic acid intake, protein intake, carbohydrate intake & hypertension	Multivariable Logistic Regression	Higher Caloric Intake, OR: 1.48 (1.15–1.92)
Cundiff, 2005	Risk	Prospective	8	Caloric Intake	1412	Progression	NIL	Spearman Correlation	Calories in kcal against DR progression rate, r = 0.07 (p = 0.007)
Alcubierre, 2016	NS	Case-Control	10	Caloric Intake	Case: 146 Ctrl: 148	Prevalence	Age, gender, diabetes duration, energy intake, educational level, physical activity, waist circumference, systolic BP, HDL Cholesterol and Diabetes treatment	Multivariable Logistic Regression	Highest energy intake tertile (T3) vs lowest energy intake tertile (T1), OR: 0.73 (0.37–1.46)

#### Fish

Two prospective studies reported a protective association between oily fish intake and DR (Table 4). Sala-Vila and associates[45] reported a decrease in risk of incident DR between those who consumed two or more weekly servings of oily fish and those who did not (HR: 0.41, 0.23–0.72). Similarly, Millen and colleagues[9] found a protective effect on DR in dark (oily) fish (consume dark fish >1 times a week vs. <1 times, OR, 95% CI: 0.32, 0.14–0.78), but not in white fish (OR, 95% CI: 1.16, 0.70–1.92).

#### Alcohol

All cross-sectional studies[40, 46, 48, 52] have reported an independent protective association between light to moderate alcohol intake and prevalence of DR, even in multivariable logistic regression models (Table 4). However, a prospective study by Young and associates[54] found a risk association between alcohol and DR, but only in those with heavy alcohol intake (heavy vs. none-moderate, relative risk, 95% CI: 2.24, 1.15–4.42). In contrast, three prospective studies and a case-control study all reported no significant associations between alcohol and DR[13, 47, 50, 53].

#### Other beverages

Limited studies–only one study per beverage—have been conducted on the associations of other beverages with DR (Table 4). A case-control study by Ma and associates[17] reported a protective effect of green tea consumption on prevalent DR (consumers vs. non-consumers, OR, 95% CI: 0.48, 0.24–0.97). Respective studies by Kumari and associates, and Millen and colleagues found no significant associations between coffee and milk with DR[9, 49].

### Associations between broader dietary patterns / characteristics and DR

#### Mediterranean (Med) diet

Evidence from an interventional study by Diaz-Lopez and associates[19] suggests a protective association of a Med diet on incident DR (Table 4). 3614 patients with type 2 diabetes from the PREDIMED trial were split between a control (low-fat) diet, and two types of Med diets. Using a multivariable cox regression model, a protective effect of the Med diet on incident DR was found (any Med diet vs. control diet, HR, 95% CI: 0.60, 0.37–0.96).

#### Total caloric intake

While the case-control study by Alcubierre and associates[39] reported no significant relationship between high caloric intake as a whole and DR (Table 4), two prospective studies by Cundiff and colleagues, and Roy and associates (OR, 95% CI: 1.49, 1.15–1.92) both reported risk associations between a high total caloric intake and DR[10, 13].

## Discussion

In our broad-based systematic review of the relationship between dietary intake and DR, a majority of studies found that intake of dietary fibre, oily fish, and a Med diet were protective of DR. In contrast, sodium and carbohydrates were not associated with DR, while high total caloric intake may be associated with greater DR risk. Importantly, the relationship between DR and several common dietary components including antioxidants, fatty acids, proteins, alcohol, and beverages, such as tea and coffee remained unclear, suggesting that more research, including longitudinal studies, are required to better understand these relationships. Our study may contribute to DR-specific dietary recommendation and complement existing DR management guidelines.

Our review provides evidence of a protective effect of dietary fibre, fruits and vegetables, and oily-fish on DR, consistent with the current literature[9, 12–16, 41]. Most fruits and vegetables are low-glycemic index foods rich in antioxidants and dietary fibre.[59] The ingestion of dietary fibres tends to modulate the postprandial glucose response[60], and is thus proposed to reduce glucose-induced damage to the retina[15]. Likewise, antioxidants are proposed to decrease oxidative stress in the retina[61], though till date there exists no clear association between antioxidants and DR (discussed later). Oily-fish is a rich source of Vitamin D and LCω3PUFAs, and it is proposed that the immune-modulatory and anti-angiogenic properties of these nutrients may play a role in the inhibition of DR[62–64]. However, it should be noted that Millen and associates found the protective effects of oily fish on DR to be independent of LCω3PUFA intake[9], suggesting these protective effects may stem from more than just the presence of LCω3PUFA’s in oily fish alone, with further research needed to confirm the exact underpinning mechanisms.

Our finding that a Med diet is protective for DR is similarly unsurprising as it is recognized as one of the healthiest dietary patterns[65–68], with several components of the Med diet, including olive oil, red wine, fibre and cereals proposed to alleviate pathogenic factors of diabetic microvascular complications such as inflammation, oxidative stress, and insulin resistance[69]. However, given that only one study has focused on the Med dietary pattern and DR, with relatively low number of incident DR cases (n = 74), our results should be interpreted with caution[19].

Alternatively, our finding that a high total caloric intake may increase the risk of DR incidence and progression[10, 13] concurs with experimental and clinical evidence suggesting that higher caloric intake increases the metabolic burden and oxidative stress in persons with diabetes, and may increase the risk of developing DR in the oxidative stress-susceptible retina[70–73]. Interestingly, evidence unequivocally suggests that there is no significant association of increased carbohydrate intake, one of the key contributors to total caloric intake, with DR[10, 43]. In fact, two studies reported protective associations between carbohydrate intake and DR;[13, 14] however, these results should be viewed with caution, as they did not adjust for important confounders such as duration of diabetes, insulin use, the quality of carbohydrates (e.g. high vs low glycemic index). In spite of a lack of significant association with DR, the monitoring of carbohydrate intake is still important for improving postprandial glucose control in patients with diabetes[74]. However, greater focus on the *quality* of carbohydrates (consumption of low-glycemic index foods), and on reducing total caloric intake, may be more beneficial in preventing the development and progression of the disease[75, 76].

While prior experimental studies have suggested a protective association between antioxidants and DR[77, 78], we found a lack of consensus over the effects of Vitamin C, E and carotenoid intake on DR in humans, similar to a review by Lee and associates[22]. Additionally, while experimental studies have also suggested PUFA intake to be protective against DR[79–81] through anti-inflammatory and anti-angiogenic properties[82], current evidence remains inconclusive[10, 12, 39, 45]. Our findings also support those of a recent meta-analysis by Zhu and associates that could not confirm a protective effect of alcohol consumption on DR[83]. While moderate alcohol consumption has been postulated to be protective of DR through multiple proposed mechanisms, including improving increasing insulin sensitivity[84] and decreasing platelet aggregability[21], such protective associations have only been reported in cross-sectional studies, with longitudinal studies reporting no clear associations. Lastly, research on the effect of popular beverages such as coffee, tea, milk[9, 17, 49] on DR remains limited, with only one study on each beverage, and no data available on the effects of soft drinks on DR. Given that the above dietary factors constitute a large component of diet, future large-scale prospective studies are warranted to elucidate their impact on DR incidence and progression.

Our findings generally support existing American Diabetic Association (ADA) guidelines for overall diabetes management that acknowledge the beneficial effects of a Med diet[7], encourage people with diabetes to consume a diet rich in fruits and vegetables[7], and recommend lower caloric intakes[7, 8]. The ADA likewise states insufficient evidence for the benefits of antioxidant supplementation[74], or to recommend an ideal amount of protein intake[85] for diabetic individuals. For other dietary components however, while we found no conclusive evidence to suggest that increased sodium and carbohydrate intake have a detrimental effect on DR risk, the ADA still recommends patients with diabetes to monitor their sodium and carbohydrate intake[85]. Likewise, while the evidence for the effect of MUFA / PUFA intake, and moderate alcohol intake on DR remains inconclusive, the ADA does recommend PUFAs and MUFAs as substitutes for saturated or trans fat[74], and also recommends moderate alcohol consumption[7] (should persons wish to drink) for patients with diabetes. The findings of our review are meant to complement and should be viewed in conjunction with the existing dietary guidelines for overall diabetes management.

While the majority of the studies included in our review had sound methodological and study qualities (with high NOS scores), there remain several restrictions in the current literature on the relationship between dietary intake and DR that limits our ability to derive more conclusive outcomes. First, most studies used FFQs to assess dietary intakes, and these questionnaires were administered only once at study baseline. While FFQs are widely used as the primary tool of dietary assessment in epidemiological studies, they also have limitations due to recall bias, and subjectivity across individuals and time-frames[86]. Future studies using more objective measures of diet such as food consumption records or which collect data across multiple time-points will provide more robust measurements of dietary intake. Second, most studies are cross-sectional, which limits their ability to establish a causal relationship between dietary factors and DR. As such, more longitudinal studies are hence warranted. Third, most studies only assessed a single dietary component or nutrient, and did not consider a broader concept of dietary intake, which is often a combination of many meals, foods and nutrients. Rather than continued focus on single nutrients, studies should also place emphasis on foods, beverages or even dietary patterns, to better reflect real world consumption habits which can be translated into clearer dietary guidelines[87, 88]. Forth, research regarding the impact of dietary intake on DME remains sparse, and future research is needed to better understand the mechanisms of diet in those with DME which may differ from DR. Lastly, it should be noted that many studies did not differentiate between type 1 and type 2 diabetes. This is important because there are pathophysiological, etiological, epidemiological and disease management differences between diabetes types, all of which may influence on the effect of dietary intake on DR and DME. Future studies should clearly identify diabetes types and provide specific data for each.

There are several strengths in our systematic review. Firstly, we sought to specifically evaluate dietary intake exposures and DR within human subjects, rather than including experimental, bio-mechanism or bio-marker studies, which allows for a more direct translation of results into dietary recommendations for patients. Secondly, as included studies were conducted on a wide variety of populations (over more than 10 different countries), this increases the generalizability of our results. However, there are also certain limitations to our study. For example, the methodological diversity across studies in assessing dietary intake exposures, and DR outcomes may affect their comparability. For example, studies using only fundus examinations, two-field or non-mydriatic fundus photography may have underestimated the number of DR cases, compared to studies using stereoscopic 7-field fundus photographs (the reference standard to detect DR as defined by the ETDRS)[29]. We were also unable to conduct a formal meta-analysis to synthesize overall findings, as within each dietary component there were few studies sufficiently similar in design, outcome, and exposure measurements to be suitable for meta-analysis.

In conclusion, our systematic review demonstrates that diet can form a crucial aspect of DR prevention and management, with evidence suggesting that dietary fibre, oily fish, and a Med diet are protective of DR, while a higher caloric intake was associated with greater DR risk. These findings may enable clinicians to make evidence-based dietary recommendations when counseling patients with diabetes who are at risk of DR. However, further prospective studies and experimental models to untangle the effects of other key dietary components on DR, such as antioxidants, fatty acids, proteins, alcohol and popular beverages, are needed in order to better inform clinical guidelines.

## Supporting information

S1 File Study quality evaluation. (XLSX)

S2 File PRISMA checklist. (DOC)
